# Evaluation of global and intragenic hypomethylation in colorectal adenomas improves patient stratification and colorectal cancer risk prediction

**DOI:** 10.1186/s13148-021-01135-0

**Published:** 2021-08-09

**Authors:** Carla Debernardi, Laura Libera, Enrico Berrino, Nora Sahnane, Anna Maria Chiaravalli, Cristiana Laudi, Mattia Berselli, Anna Sapino, Fausto Sessa, Tiziana Venesio, Daniela Furlan

**Affiliations:** 1 Pathology Unit, Candiolo Cancer Institute, FPO-IRCCS, Candiolo, Italy; 2 Pathology Unit, Department of Medicine and Surgery, University of Insubria, Varese, Italy; 3 Department of Medical Sciences, University of Torino, Torino, Italy; 4 Pathology Unit, ASST Sette Laghi, Varese, Italy; 5 Gastroenterology, Candiolo Cancer Institute, Candiolo, Italy; 6 Surgical Oncology and Minimally Invasive Unit, Department of Surgery, ASST Sette Laghi, Varese, Italy; 7 Research Center for the Study of Hereditary and Familial Tumors, Department of Medicine and Surgery, University of Insubria, Varese, Italy

**Keywords:** Colorectal adenomas, LINE-1 hypomethylation, L1-*MET*, Bisulfite pyrosequencing, CRC risk

## Abstract

**Background:**

Aberrant DNA hypomethylation of the long interspersed nuclear elements (LINE-1 or L1) has been recognized as an early event of colorectal transformation. Simultaneous genetic and epigenetic analysis of colorectal adenomas may be an effective and rapid strategy to identify key biological features leading to accelerated colorectal tumorigenesis. In particular, global and/or intragenic LINE-1 hypomethylation of adenomas may represent a helpful tool for improving colorectal cancer (CRC) risk stratification of patients after surgical removal of polyps. To verify this hypothesis, we analyzed a cohort of 102 adenomas derived from 40 high-risk patients (who developed CRC in a post-polypectomy of at least one year) and 43 low-risk patients (who did not develop CRC in a post-polypectomy of at least 5 years) for their main pathological features, the presence of hotspot variants in driver oncogenes (*KRAS, NRAS, BRAF* and *PIK3CA*), global (LINE-1) and intragenic (L1-*MET*) methylation status.

**Results:**

In addition to a significantly higher adenoma size and an older patients’ age, adenomas from high-risk patients were more hypomethylated than those from low-risk patients for both global and intragenic LINE-1 assays. DNA hypomethylation, measured by pyrosequencing, was independent from other parameters, including the presence of oncogenic hotspot variants detected by mass spectrometry. Combining LINE-1 and L1-*MET* analyses and profiling the samples according to the presence of at least one hypomethylated assay improved the discrimination between high and low risk lesions (*p* = 0.005). Remarkably, adenomas with at least one hypomethylated assay identified the patients with a significantly (*p* < 0.001) higher risk of developing CRC. Multivariable analysis and logistic regression evaluated by the ROC curves proved that methylation status was an independent variable improving cancer risk prediction (*p* = 0.02).

**Conclusions:**

LINE-1 and L1-*MET* hypomethylation in colorectal adenomas are associated with a higher risk of developing CRC. DNA global and intragenic hypomethylation are independent markers that could be used in combination to successfully improve the stratification of patients who enter a colonoscopy surveillance program.

**Graphic abstract:**

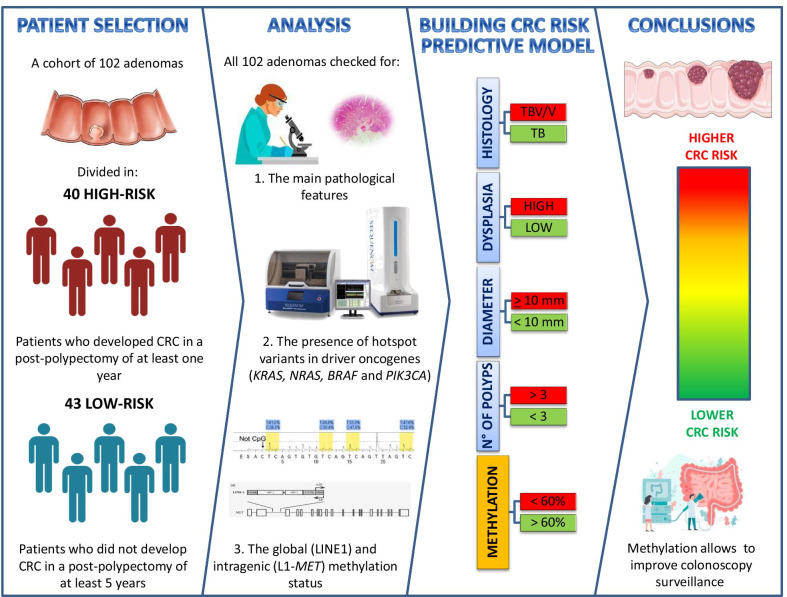

**Supplementary Information:**

The online version contains supplementary material available at 10.1186/s13148-021-01135-0.

## Background

Hypomethylation preferentially affects the DNA repetitive sequences, such as long interspersed nuclear elements (LINE-1s or L1s) that, being 17% of the human genome, can be considered a reliable surrogate measure of global DNA hypomethylation [1]. More than 2000 of these LINE-1 elements are intragenic and can modulate the expression of their host genes as cis-regulatory elements [2]. Among L1 host genes potentially involved in cancer, *MET* oncogene is of particular interest as its aberrant activation contributes to tumor onset, progression and metastasis of different types of solid tumors, including carcinomas [3]. Demethylation of its LINE-1 sequence, located in the second intron, was demonstrated to drive the transcription of a chimeric isoform associated with cancer phenotype, LINE1-*MET* or L1-*MET* [4–8].

Along with the onset of oncogenic mutations [9], LINE-1 hypomethylation was recognized as an important feature of early-onset colorectal cancer (CRC) and a prognostic biomarker for CRCs with distinct molecular phenotypes [10–14]. LINE-1 hypomethylation has been associated with colorectal cancer progression [15, 16] and, more recently, with the transition from normal mucosa to adenomas, the colorectal precancerous lesions [17]. At this regard, Jiang et al*.* (2017) correlated LINE-1 hypomethylation of adenomas with the presence of synchronous CRC [18] and we reported that patients affected by *MUTYH*-Associated Polyposis (MAP), a hereditary colorectal cancer disease, exhibit a high frequency of both LINE-1and L1-*MET* hypomethylated adenomas [19]. Moreover, Shademan et al*.* [20] showed that LINE-1 promoter methylation in advanced adenomas was significantly lower than that in non-advanced adenomas.

As CRC develops mainly via the adenoma–carcinoma sequence [21], endoscopic resection of adenomas has become an important tool to significantly reduce CRC incidence and mortality. This has led to recommend endoscopic screening for stratifying patients according to their CRC risk [22] and for planning surveillance program whose intervals rely on the pathological features of the resected adenomas [23, 24]. Although epidemiologic studies have shown the efficacy of endoscopic screening [23], colonoscopy monitoring has some strong limitations that can considerably affect its effectiveness: (i) the intervals are defined according to CRC-risk guidelines considering as main criteria the number and size of the removed polyps but lacking information on important molecular features, (ii) colonoscopy is an invasive procedure [25], (iii) patients show a low adherence to the surveillance program, and (iv) this type of screening is associated with high costs for the National Health Systems (NHS). Therefore, alternative or supportive biomarkers able to reduce unnecessary invasive procedure and predict recurrence are highly required [26].

In order to assess whether global and/or intragenic LINE-1 hypomethylation of adenomas may represent a helpful tool for CRC risk stratification of patients after surgical removal of polyps, we evaluated the level of globally and intragenic LINE-1 in 102 adenomas derived from a selected pilot cohort including 40 patients who had developed CRC after the adenoma onset (high-risk patients) and 43 patients who had not developed cancer (low-risk patients).

## Results

### Clinical pathological features and mutational status of the adenoma cohort

**Fig. 1:**
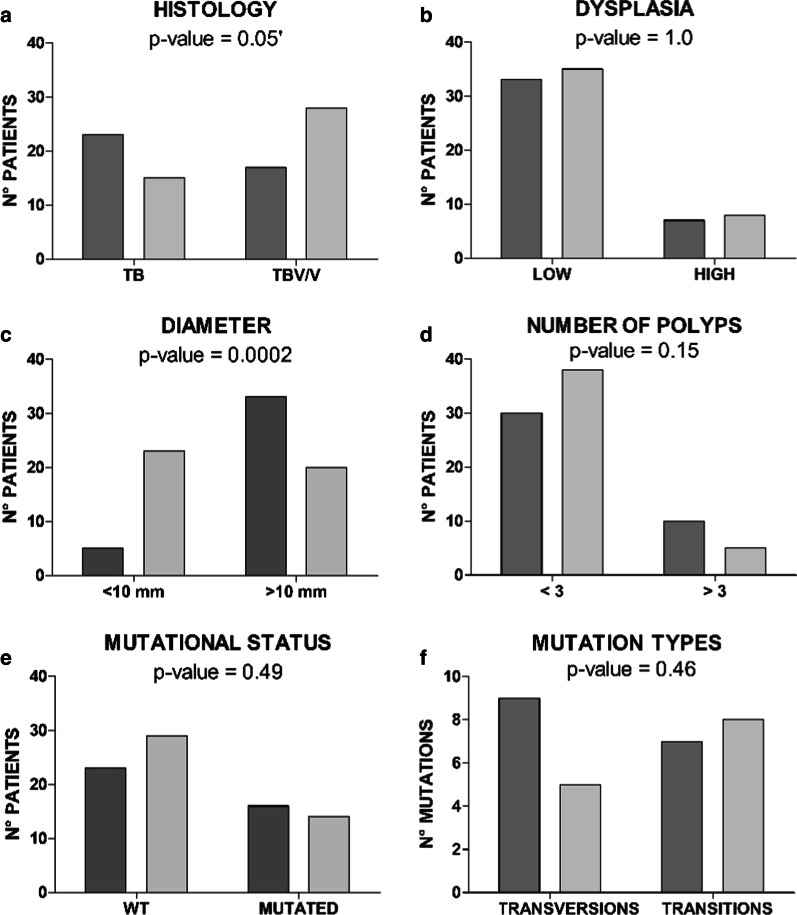
Adenoma clinical and pathological features. **a** Adenoma histology: tubular adenomas (TB) versus tubulovillous/villous adenomas (TBV/V). **b** Adenoma dysplasia: low dysplasia grade versus high grade. **c** Adenoma diameter (mm): the adenomas were classified in two groups, using as a threshold 10 mm, a parameter currently adopted to identify higher CRC risk patients. **d** Number of polyps per patient: patients were divided using as a threshold 3 adenomas removed at the same time, a parameter currently used to identify higher CRC risk patients. **e** Adenoma mutational status: wild-type (WT) versus mutated adenomas. **f** Mutations identified analyzing adenomas were classified in transversions or transitions. High-risk group (dark gray) and Low-risk group (light gray)

High- and low-risk cases were compared for their main clinical and pathological features. The high-risk patient group was characterized by a significantly higher adenoma size (diameter: 17.2 vs 9.7 mm; *p* = 0.0002), an older patient age (69.8 vs 63.9 years; *p* = 0.01), and a slight prevalence of tubular morphology (57.5% vs. 34.9%) that resulted near significant (*p* = 0.05). (Table 1 and Fig. 1). On the contrary, no differences were found considering the presence and the number of synchronous polyps, the right or left colon localization, and the dysplasia grade.

**Table 1:** Population/sample description

Cases characteristics	No	Mean (SD) or %	CONTROLS characteristics	No	Mean (SD) or %	*p*-value*
Age (years)	40	69.8 (± 7.6)	Age (years)	43	63.9 (± 9.8)	0.01*
Sex			Sex			0.51
Female	15	37.5%	Female	20	46.5%	
Male	25	62.5%	Male	23	53.5%	
Hystology			Hystology			0.05
TB	23	57.5%	TB	15	34.9%	
TBV/V	17	42.5%	TBV/V	28	65.1%	
Dysplasia Grade			Dysplasia Grade			1.0
High	7	17.5%	High	8	18.6%	
Low	33	82.5%	Low	35	81.4%	
Diameter (mm)	38	17.2 (± 9.0)	Diameter (mm)	43	9.7 (± 6.7)	0.0002***
No Polyps	40	1.7 (± 0.9)	N° Polyps	43	1.6 (± 1.0)	0.15
Localization			Localization			0.63
Right	15	37.5%	Right	15	34.9%	
Left	25	62.5%	Left	27	62.8%	
Year Adenoma	40	2008 (± 4.1)	Year Adenoma	43	2007 (± 4.8)	0.89
Year CRC	40	2013 (± 2.6)	Year FOLLOW-UP	43	2018	–
Mutational status			Mutational Status			0.49
Wild Type	23	57.5%	Wild Type	29	67.4%	
Mutated	16	40.0%	Mutated	14	32.6%	
Transversions	9	56.2%	Transversions	5	35.7%	0.46
Transitions	7	43.7%	Transitions	8	57.1%	
L1 Methylation			L1 Methylation			
Global	40	58.9 (± 5.3)	Global	43	60.9 (± 4.4)	0.05
Intragenic	29	58.5 (± 2.3)	Intragenic	43	60.0 (± 4.5)	0.14
L1 Methylation			L1 Methylation			
Global (≤ 60%)	24	60.0%	Global (≤ 60%)	19	44.2%	0.19
Intragenic (≤ 60%)	23	79.1%	Intragenic (≤ 60%)	24	55.8%	0.05

SD = standard deviation, TB = tubular, TBV = tubulovillous, V = villous

^*^pvalue were obtained by Mann–Whitney Test for continuous variables and by Fisher Test for categorical variables

The two adenoma cohorts were analyzed for the presence of hotspot pathogenic variants in the most frequently mutated CRC oncogenes (*KRAS, NRAS, BRAF* and *PIK3CA*), but no significant differences were found (Fig. 1). Pathogenic variants were identified in 16/40 (40%) of high-risk cases and in 14/43 (32.6%) of the low-risk cases (Table 1). These alterations were mostly *KRAS* variants (25/30; 83.3%), prevalently located on codon 12 (17/30; 56.7%), with *KRAS* p.G12D (6/30, 20%) and *KRAS* p.G13D (7/30, 23.3%) being the most common substitutions. Two high-risk cases were distinguished by the presence of *BRAF* p.V600E and *PIK3CA* p.E545K mutations, whereas one adenoma of the low-risk cases showed *NRAS* p.G12D (Additional file 1: Table S1). Overall, the frequency of transversions (G > T; G > C; T > A) and transitions (G > A) was comparable in the two cohorts, although in the high-risk case subset reported a higher number of transversions (56.2 *vs* 35.7%) (Table 1 and Fig. 1).

### DNA hypomethylation profile for global and intragenic LINE-1

**Fig. 2:**
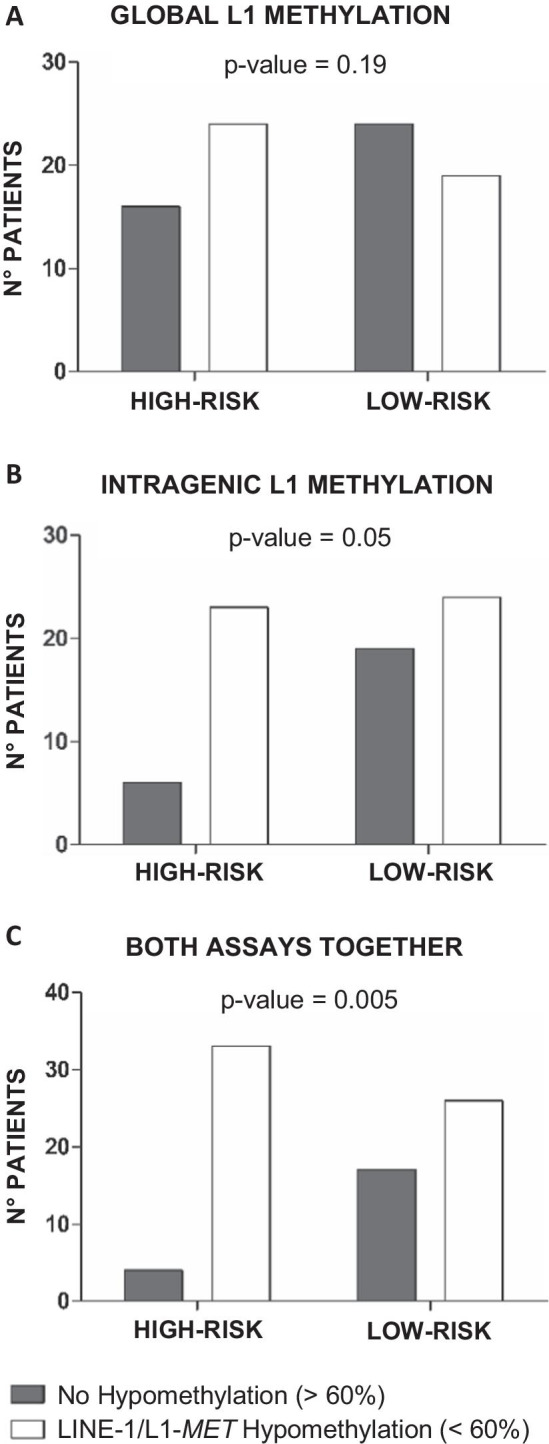
Methylation assays in high-risk and low-risk patients. Adenomas were considered non-hypomethylated (in dark gray) when assays showed methylation levels above 60%. Adenomas were considered hypomethylated (in white) when assays displayed methylation level below 60%. **a** Global L1 methylation assay. **b** Intragenic L1-*MET* methylation assay. **c** Combination of the two methylation assays: we consider hypomethylated patients with at least one assay below 60%

Global DNA and intragenic hypomethylation were investigated by carrying out LINE-1 and L1-*MET* assays on all the adenomas. Global LINE-1 study was possible in adenomas of all the 83 patients, whereas L1-*MET* methylation analysis was successful for 72 of the 83 (87%) patients. The methylation levels were evaluated both as a discrete and a continuous variable. In line with our previous reports [14, 15], we used 60% as a methylation threshold. High-risk cases were more hypomethylated than the low-risk (24/40 high-risk cases *vs* 19/43 low-risk cases for global LINE-1 assay; 23/29 high-risk cases *vs* 24/43 low-risk cases for L1-*MET* assay). Similar results were found using percentages of methylation as a continuous variable (58.9% *vs.* 60.9% for global LINE-1 assay and 58.5% *vs*. 60.0% for L1-*MET* assay) (Table 1). For both analyses, we observed a suggestive statistical trend (Table 1 and Fig. 2a, b).

Remarkably, combining the results of LINE-1 and L1-*MET* analyses and considering as hypomethylated all the samples with at least one assay < 60%, only 4 patients of high-risk group had no hypomethylated adenomas, whereas in the low-risk group 17 patients showed the same status (*p* = 0.005) (Fig. 2c). In detail, in the high-risk group, 14 (35%) patients exhibited adenomas with hypomethylation on both markers, 10 (25%) had hypomethylation only for LINE-1 and 9 (22.5%) only for L1-*MET*. By contrast, in the low-risk set the samples with concordant hypomethylation were 17 (39.5%), while those with only LINE-1 or L1-*MET* demethylation were 2 (4.6%) and 7 (16.3%), respectively (Additional file 1: Table S1). In both subsets, the methylation status was unrelated to the presence of pathogenic variants. Moreover, tubular adenomas of the high-risk cases showed a mean methylation level under 60% (L1 = 59.7, L1-*MET* = 59.4), whereas tubular adenomas of the low-risk patients preferentially displayed a mean methylation level greater than 60% (L1 = 61.7, L1-*MET* = 61.4) (Additional file 2: Fig. S1).

**Fig. 3:**
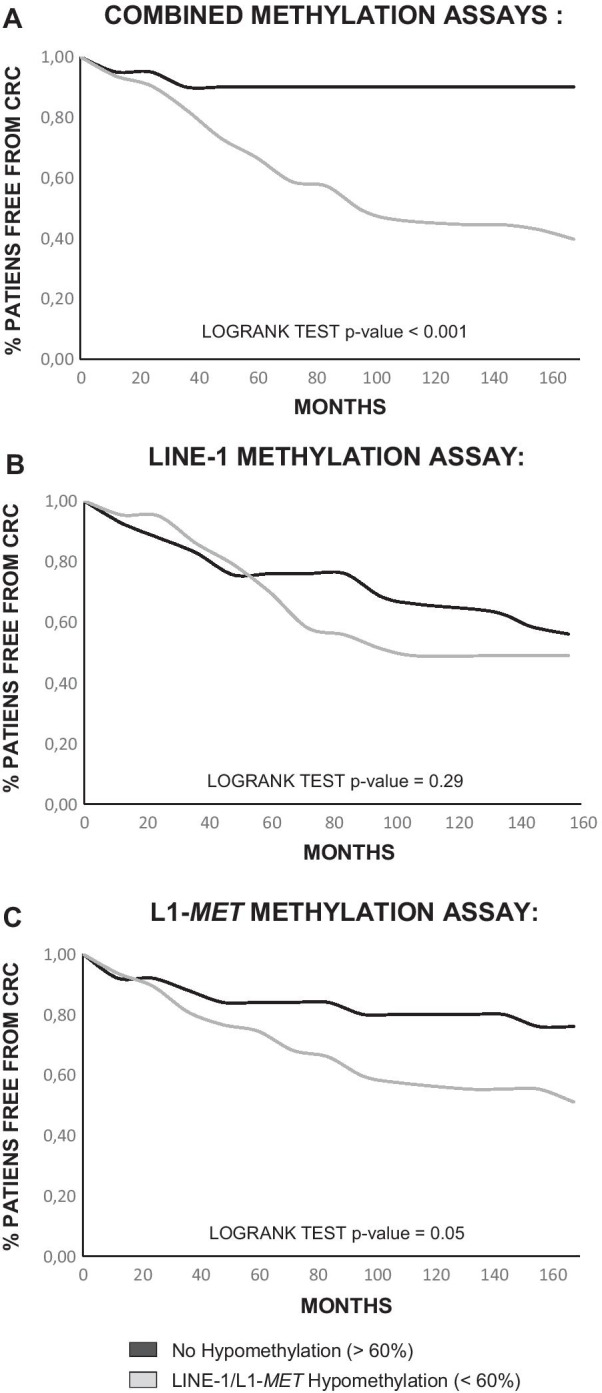
Methylation level and time free from CRC. Percentage of patients who had not developed CRC is reported on Y-axis, whereas on X-axis time is represented in months. Gray curve shows patients with hypomethylated adenomas, and black curve displays patients with non-hypomethylated adenomas. **a** Hypomethylation was assessed when at least one of the two assays was below 60%. **b** Hypomethylation according to only the LINE-1 assay below 60%. **c** Hypomethylation according to only the L1-*MET* assay below 60%

The presence of at least one methylation assay < 60% provided the patients with a significantly (*p* < 0.001) higher risk of developing CRC after the adenoma onset (Fig. 3a). In particular, L1-*MET* assay displayed a higher, near significant, sensitivity in discriminating patients at risk of developing CRC than LINE-1 assay (*p* = 0.05 *vs. p* = 0.29) (Fig. 3b, c).

### Logistic models

The adenoma cohort was first analyzed by using a model containing the parameters currently used to assess higher CRC risk patients, namely tubulovillous/villous (TBV/V) histology, high grade of dysplasia, > 10 mm diameter, and > 3 simultaneous number of polyps (MOD1). In order to verify if the methylation status could improve CRC risk prediction, all cases were reclassified by adding this variable (at least one methylation assay < 60%) to the model (MOD2).

**Fig. 4:**
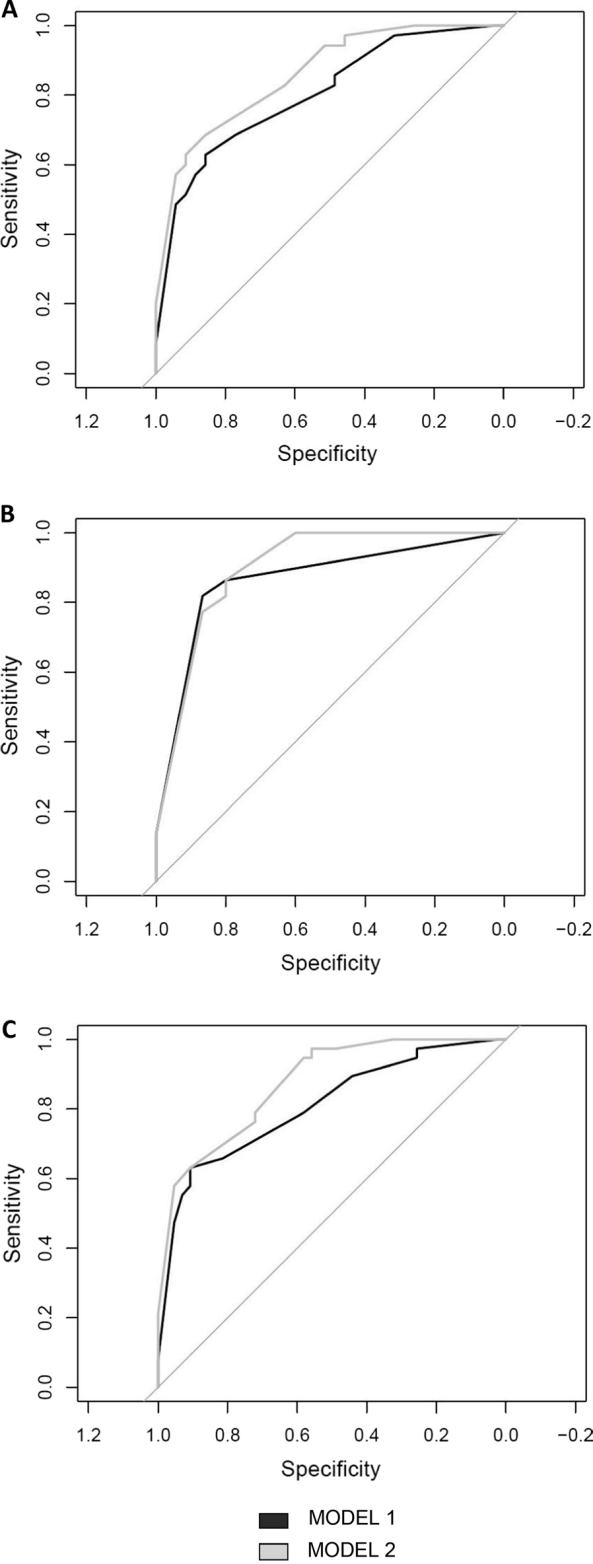
ROC curves representing the logistic models to identify higher CRC risk patients**.** MOD1 is the logistic model without the methylation parameter, while MOD2 comprises the methylation value. Black curves show the efficiency of the models built using the current parameters, while gray curves display the efficiency of the new models comprising the methylation level. **a** All adenomas of the study were evaluated. **b** The subset consisting only of tubular adenomas was considered. **c** The subset including only the controls with at least 5 years of follow-up and the cases that had developed CRC within 10 years was assessed

The difference between MOD1 and MOD2 was evaluated by ROC curves. MOD2 turned out to be more accurate than MOD1 as shown by the ROC curves (AUC 1 = 0.81[0.71–0.90] *vs* AUC2 = 0.87 [0.80–0.94]) and the DeLong test (*p* = 0.02) (Fig. 4a). By applying the standard guidelines (MOD1), 4/40 (10%) of the cases were lost as higher CRC risk patients, whereas the addition of the methylation parameter (MOD2) allowed to reduce the misclassification to 1/40 (2.5%), thus improving the effectiveness of surveillance after adenoma removal.

Data were then verified for the presence of sample bias potentially affecting the logistic model results. As the high-risk cases had a number of tubular adenomas higher than tubulovillous/villous (TBV/V) adenomas, the logistic model was re-analyzed considering only the tubular adenomas in the two groups.  The ROC curves of both models improved, although the difference between their underlying areas was not significant (AUC2 = 0.93 [0.85–1.00] *vs.* AUC1 = 0.91 [0.83–1.00]; *p* = 0.57) (Fig. 4b).

The samples were collected between 1998 and 2015, with a follow-up range of 1–14 years (mean 5.5 years). In order to test if time could influence the models, we selected a subset including only controls with at least 5 years of follow-up and cases that had developed CRC within 10 years. The resulting ROC curves were not affected by time and, although the difference was not significant, the model including the methylation variable still had a larger AUC than Model 1 (AUC1 = 0.81 [0.71–0.90] vs AUC 2 = 0.86 [0.77–0.94], *p* = 0.18) (Fig. 4c).

## Discussion

Most of the patients entering in colonoscopy screening programs develop at least one adenoma which may eventually transform into a cancer over time [27]. In this regard, present surveillance programs, based on size, morphology, and absence of synchronous polyps, frequently result in unnecessary repeated colonoscopy. This implies the need of identifying novel noninvasive, selective, and reliable markers for improving the stratification and the follow-up of those specific patients at higher CRC risk [26].

Global DNA hypomethylation, measured by LINE-1, was previously recognized as a molecular feature able to confer a higher predisposition to the development of colorectal adenomas and cancers [9, 20]. Jiang and colleagues reported that LINE-1 hypomethylation increased in the transition from normal mucosa to CRC and could be used to predict the onset of synchronous CRCs [18]. In addition, the specific role of LINE-1 methylation in the transition from normal mucosa to adenoma was proved by a genome-wide expression analysis based on the comparison of normal adjacent, adenomatous and CRC tissues [17]. Overall, the number of studies testing the involvement of epigenetic regulatory mechanisms in colorectal adenoma–carcinoma transition is rapidly growing [20, 28, 29].

In the present retrospective study, we demonstrate, for the first time, that in colorectal adenomas the combined evaluation of global genomic and intragenic hypomethylation, analyzed by LINE-1 and L1-*MET* assays, could be used as a potential molecular marker to improve the classification of patients for their CRC risk.

Differently from previous reports, we here assessed not only the global DNA methylation in adenomas, but also the methylation level of an intragenic locus specific sequence located on chromosome 7, L1-*MET*. This approach improved the sensitivity of the analysis. As a matter of fact, most of the samples displayed concordant methylation, but some of them were hypomethylated for only one of the two analyzed markers. In this event, L1-*MET* resulted more frequently hypomethylated and powerful in discriminating higher CRC risk patients than LINE-1. Xu and colleagues, performing an unsupervised analysis on normal mucosa, non-CIMP and CIMP CRCs, described differential and specific DNA methylation sites along all chromosomes [30]. In agreement, Timp et al. demonstrated that large (hundreds of kb) hypomethylated blocks are scattered throughout the genome and characteristically associated with colorectal cancers [29]. According to these authors, the hypomethylated blocks acquire an early dysregulation in the CRC progression, already at the stage of premalignant lesions. Moreover, the genes enriched of hypomethylated stretches show a high inter-individual expression variability [29]. Indeed, though LINE-1 sequences are highly represented throughout the human genome, we can hypothesize that L1-*MET* locus is harbored in a hypomethylated block and might specifically gain an early abnormal epigenetic status. However, in order to definitely assess the detailed differences between LINE-1 and L1-*MET* markers in predicting the hypomethylation profile of colon premalignant lesions, an increased number of observations will be necessary.

In this study, DNA hypomethylation status was independent of the other CRC risk factors currently used to classify adenomas. High-risk cases were more hypomethylated than low-risk cases and profiling the samples according to the presence of at least one assay of LINE-1/ L1-*MET* below 60% improved the discrimination between high and low risk lesions. In agreement with former studies [31], our multivariate analysis confirmed that histology, grade of dysplasia, polyp diameter, polyp number and follow-up time were independent prognostic factors. However, the addition of the methylation parameter resulted in a higher accuracy of the CRC risk prediction, reducing misclassification of high-risk adenomas from 10 to 2.5%.

These results were confirmed when only tubular histology or a longer time between polypectomy and CRC onset were considered. Generally, it takes 5–10 years for a small adenoma to transform into a cancer [32].

Our cohort was slightly enriched for the presence of TBV/V adenomas that were more frequent in the low-risk compared to high-risk subset. This unbalance was due to the need of including at least 25 mm^2^ sized samples to properly carry out the molecular analyses. TBV/V histology is generally correlated with a larger size of adenomas. However, as previously reported [18], hypomethylation levels were not associated with villous morphology. Similarly, LINE-1/L1-*MET* hypomethylation was unrelated to adenoma size and to patient age, suggesting that DNA hypomethylation is independent of these variables.

As expected [33, 34], most of the mutations found in our study occurred in *KRAS* gene, whereas *BRAF* and *PIK3CA* variants were detected in only two cases. No differences in the distribution and in the type of the variants were identified in the two cohorts, though transversions were more frequently found in cancer-associated adenomas. In line with our previous report [19], LINE-1/L1-*MET* methylation levels were unrelated to the presence of hot-spot mutations in *KRAS* gene. Accordingly, Luo and colleagues, in a comprehensive methylome analysis of CRC progression, demonstrated that only a subset of high frequency CpG site methylated adenomas showed *KRAS* mutations [35].

Transversions on *KRAS* oncogene have been recognized as the hallmark of increased oxidative DNA damage due to the overproduction of ROS, normally involved in many redox-governing processes of the cells [36]. BER pathway is the main mechanism controlling the removal and repair of oxidized DNA bases. We showed [19] that in *MUTYH*-associated polyposis, a hereditary colorectal cancer syndrome marked by impaired BER, adenomas exhibit a pronounced hypomethylation. Oxidative stress may drive the first steps of colorectal tumorigenesis by two independent pathways, one involving specific oncogenic mutations and the other one involving DNA methylation alterations.

Despite the novelty of our results, we are aware of some limitations. Mostly, this was a pilot study carried out on a limited retrospective cohort. In order to reduce the potential sample bias due to this limitation, we reanalyzed the logistic models taking into account histology and time. However, the present results need to be confirmed increasing the number of observations in a prospective study. Secondly, the efficiency of LINE-1 and L1-*MET* assays is different as L1-*MET* is a single-copy sequence, whereas LINE-1 represents up to 17% of the genome. This difference can be particularly evident when small amount of formalin-fixed, paraffin-embedded (FFPE) tissue is analyzed as in the case of samples derived from colorectal adenomas. Our future aim will be the standardization of these two assays by using alternative technical approaches to pyrosequencing, such as next generation sequencing (NGS). This will allow a higher throughput methylation analysis starting from a smaller amount of DNA.

## Conclusions

Our findings indicate that LINE-1 and L1-*MET* hypomethylation in colorectal adenomas are associated with an increased CRC risk. These preliminary results show that LINE-1 and L1-*MET* methylation evaluation are independent hypomethylation markers whose combined use could successfully improve the stratification of patients entering a colonoscopy surveillance program after surgical removal of polyps.

## Methods

### Patients

We selected 83 patients with sporadic adenomas, collected between 1998 and 2015 from the files of the Pathology Unit, Ospedale di Circolo-University of Insubria, Varese.

This cohort included 40 patients affected by CRC with polyp(s) removed at least one year before CRC diagnosis who were considered as “high-risk cases.” Other 43 patients were included as “low-risk cases” because they had undergone endoscopic polypectomy, and they never developed CRC in a post-polypectomy surveillance of at least 5 years (Additional file 1: Table S1). None of the two patient cohorts was part of an endoscopy-based colorectal cancer screening program, and all but two cases were submitted to endoscopic polypectomy.

Considerable evidence suggests that important lesions may be missed at colonoscopy and several studies have suggested that patients who develop cancer after colonoscopy are more likely to have proximal compared than distal cancers. This could be due to quality of bowel preparation, failure to fully examine the proximal colon, differences in proximal polyp/cancer morphology, the skill of the endoscopist, and variable quality of colonoscopy [37–39].

We took into consideration all these issues in the patient selection, and criteria for patient exclusions were: low quality of the intestinal preparation and known predisposing factors of CRC including familiarity for polyposis or CRC, CRC diagnosed before 55 years of age, presence of inflammatory idiopathic diseases, and occurrence of 6 or more synchronous adenomas during polypectomy.

Among high-risk cases, the mean follow-up time between polypectomy and CRC occurrence was 64 months. The minimum interval time of 12 months was observed in only six high-risk patients that developed adenomas in the left sites. Moreover, two of these patients were submitted to a surgical resection of the adenomas, while the other four patients developed CRCs in a different site than the adenoma.

The present study was carried out according to the research rules of our institutional medical ethical committees on human experimentation, and appropriate written informed consent was collected from all individuals included in the analysis.

### Selection criteria and clinical-pathological analysis of adenomas

We analyzed a total of 102 adenomas. We selected samples of adenomas of at least 25 mm^2^ and with available clinical-pathological data: site, diameter, grade of dysplasia (low or high), histology (tubular: TB; or tubulovillous: TBV; or villous: V), number of synchronous adenomas removed during colonoscopy (never more than six adenomas), age and sex of the patients, occurrence of a CRC during a post-polypectomy surveillance of at least 5 years. For 15 patients, two or more adenomas were analyzed and for the statistical analyses we selected the adenomas with higher CRC risk factors, including villous architecture, higher grade of dysplasia and larger diameter. Moreover, when two adenomas had the same clinical-pathological characteristics, we selected the one with the lowest methylation value. After this screening, we carried out the analyses on a final set of 83 adenomas, each adenoma representing one single patient. Data concerning all the analyzed adenomas are reported in Additional file 1: Table S1.

### Gene mutation analysis

Molecular analyses were performed on formalin-fixed, paraffin-embedded (FFPE) tissue sections using three representative 8-μm-thick sections of tissue samples. The pathologist selected an area with more than 50% of dysplastic cells. DNA was extracted after manual dissection using a QIAamp DNA FFPE Tissue Kit (Qiagen, Hilden, Germany) according to the manufacturer's protocol and quantified using the Qubit 2.0 Fluorometer (Life Technologies/Thermo Fisher Scientific, Wilmington, DE, USA) following the protocol of High Sensitivity DNA Kit (Life Technologies, Eugene, OR, USA).

All the 102 adenomas were checked for hotspot mutations in *KRAS, BRAF, NRAS* and *PIK3CA* genes using the mass spectrometry matrix-assisted laser desorption ionization time of flight method with the MassARRAY System (Agena Bioscience, Hamburg, Germany) with Myriapod Colon Status (Diatech Pharmacogenetics, Jesi, Italy) (Additional file 1: Table S1).

### LINE-1/L1-*MET* methylation analysis

The methylation status of global and local LINE-1 sequences was evaluated by bisulfite-pyrosequencing. DNA bisulfite conversion was performed using the Epitect Kit (Qiagen, Hilden, Germany) according to the manufacturer's instructions.

As previously reported, global LINE-1 methylation status was assessed through the quantification of the mean methylation percentage of four consecutive CpG sites in the LINE-1 promoter region (GenBank Accession Number: X58075) [19]. Intragenic levels of LINE-1 methylation were analyzed using the L1-MET assay: the forward PCR primer (5′-GAGATGAATTTAGTATTTTAGATGGAAATG-3) was located inside the LINE-1 promoter, and the reverse primer (5′-biotin-ACAACTCCCATCTACAACTCCCA-3′) was designed within the *MET* gene intron between exons 2 and 3. The sequencing primer (5′-TTTAGATGGAAATGTAGAAATTAT-3′) amplified a product that includes three CpG sites whose mean methylation percentage was quantified (GenBank Accession Number: NG_0089961). Each sample was loaded two times for pyrosequencing, and fully methylated and unmethylated DNA (Millipore, Billerica, MA, USA) were used as positive and negative controls in each experiment.

In our previous studies, we carefully evaluated the methylation status of LINE-1/L1-*MET* in histologically normal colonic mucosa [14, 19]. Sahnane et al. [14] found that the average percentage of LINE-1 methylation was 64.5 ± 2% with a very low variability among different samples. Furlan et al. [19], evaluating both LINE-1 and L1-*MET*, reported an overall percentage comprised between 60–70%. On the basis of those results, we used the lowest methylation value (60%) of normal mucosa as the methylation threshold and we considered methylation percentage values both as a discrete variable and as a continuous variable (Table 1). Detailed methylation data concerning the 102 analyzed adenomas are reported in Additional file 1: Table S1

### Statistical analysis

Statistical analyses were performed using R Studio Version 1.1.442—© 2009–2018 R Studio, Inc. All p values reported are two-tailed, and values of *p* < 0.05 were considered to be statistically significant. The Shapiro–Wilk test was used to verify that data were not normally distributed, whereas the Mann–Whitney and Fisher test were applied to determine differences between the populations. We used a logistic regression to measure the association between methylation and developing CRC. In multivariable analysis, the following variables were considered: histology, grade of dysplasia, diameter of the lesions, number of synchronous adenomas, time of follow-up after polypectomy and time of CRC occurrence after polypectomy. The curves representing sensitivity of methylation in discriminating CRC risk were evaluated by the log-rank test. Differences between two ROC curves were tested by DeLong’s Test.

## Supplementary Information

**Additional file 1:** **Table 1S.** Complete dataset used for the study. Adenomas evidenced in red were excluded from the statistical analysis since they did not match the selection criteria. Abbreviations : M = male, F = female, CNTRL = control, TB = tubular, TBV = tubulovillous, V = villous, mm= millimeter, SX = left, DX = right.

**Additional file 2: Fig. S1.** Methylation level in relation to the adenoma histotype. A) LINE-1 methylation level in tubular and tubulovillous/villous adenomas divided in cases and controls (CNTRL). B) L1-MET methylation level in tubular and tubulovillous/villous adenomas divided in cases and controls (CNTRL). The dotted line indicates the methylation threshold (60%) below which adenomas are considered hypomethylated. The green lines indicate the average level of methylation of the low-risk group, while the red lines indicate the average level of high-risk group.

## Abbreviations

AUC: Area Under the ROC Curve
BER: Base Excision Repair
CIMP: CpG island methylator phenotype
CRC: Colorectal cancer
FFPE tissue: Formalin-fixed, paraffin-embedded tissue
LINE-1: Long Interspersed Nuclear Element-1
L1-*MET*: Long interspersed nuclear element-1 in the second intron of *MET* gene
MAP: *MUTYH*-Associated Polyposis
MOD1: Logistic Model 1
MOD2: Logistic Model 2
NHS: National Health Systems
ROC curve: Receiver Operating Characteristic curve
TBV/V: Tubulovillous/villous
