# Correction to: Evaluation of global and intragenic hypomethylation in colorectal adenomas improves patient stratification and colorectal cancer risk prediction

**DOI:** 10.1186/s13148-021-01166-7

**Published:** 2021-09-21

**Authors:** Carla Debernardi, Laura Libera, Enrico Berrino, Nora Sahnane, Anna Maria Chiaravalli, Cristiana Laudi, Mattia Berselli, Anna Sapino, Fausto Sessa, Tiziana Venesio, Daniela Furlan

**Affiliations:** 1 Pathology Unit, Candiolo Cancer Institute, FPO-IRCCS, Candiolo, Italy; 2 Pathology Unit, Department of Medicine and Surgery, University of Insubria, Varese, Italy; 3 Department of Medical Sciences, University of Torino, Torino, Italy; 4 Pathology Unit, ASST Sette Laghi, Varese, Italy; 5 Gastroenterology, Candiolo Cancer Institute, FPO-IRCCS, Candiolo, Italy; 6 Surgical Oncology and Minimally Invasive Unit, Department of Surgery, ASST Sette Laghi, Varese, Italy; 7 Research Center for the Study of Hereditary and Familial Tumors, Department of Medicine and Surgery, University of Insubria, Varese, Italy

## Correction to: Clin Epigenet (2021) 13:154 https://doi.org/10.1186/s13148-021-01135-0

After publication of the original article [1], the authors identified an error in some authors’ affiliations list. The incorrect affiliations list is:

Carla Debernardi^1,7^, Laura Libera^2,7^, Enrico Berrino^1,3,7^, Nora Sahnane^4,7^, Anna Maria Chiaravalli^4,7^, Cristiana Laudi^5^, Mattia Berselli^6,7^, Anna Sapino^1,3^, Fausto Sessa^2,7^, Tiziana Venesio^1,7*†^ and Daniela Furlan^2,7†^

^1^Pathology Unit, Candiolo Cancer Institute, FPO-IRCCS, Candiolo, Italy. ^2^Pathology Unit, Department of Medicine and Surgery, University of Insubria, Varese, Italy. ^3^Department of Medical Sciences, University of Torino, Torino, Italy. ^4^Pathology Unit, ASST Sette Laghi, Varese, Italy. ^5^Gastroenterology, Candiolo Cancer Institute, Candiolo, Italy. ^6^Surgical Oncology and Minimally Invasive Unit, Department of Surgery, ASST Sette Laghi, Varese, Italy. ^7^Research Center for the Study of Hereditary and Familial Tumors, Department of Medicine and Surgery, University of Insubria, Varese, Italy.

The correct affiliation is:

Carla Debernardi^1^, Laura Libera^2,7^, Enrico Berrino^1,3^, Nora Sahnane^4,7^, Anna Maria Chiaravalli^4,7^, Cristiana Laudi^5^, Mattia Berselli^6,7^, Anna Sapino^1,3^, Fausto Sessa^2,7^, Tiziana Venesio^1,*†^, Daniela Furlan^2,7†^

^1^Pathology Unit, Candiolo Cancer Institute, FPO-IRCCS, Candiolo, Italy. ^2^Pathology Unit, Department of Medicine and Surgery, University of Insubria, Varese, Italy. ^3^Department of Medical Sciences, University of Torino, Torino, Italy. ^4^Pathology Unit, ASST Sette Laghi, Varese, Italy. ^5^Gastroenterology, Candiolo Cancer Institute, FPO-IRCCS, Candiolo, Italy. ^6^Surgical Oncology and Minimally Invasive Unit, Department of Surgery, ASST Sette Laghi, Varese, Italy. ^7^Research Center for the Study of Hereditary and Familial Tumors, Department of Medicine and Surgery, University of Insubria, Varese, Italy.

The author group has been updated above and the original article [1] has been corrected.
