# Supplementary material for: Evaluation of global and intragenic hypomethylation in colorectal adenomas improves patient stratification and colorectal cancer risk prediction
Source: Clin Epigenetics. 2021 Aug 9;13:154. doi: 10.1186/s13148-021-01135-0 (PMC8351348; doi:10.1186/s13148-021-01135-0)
Supplement: Supplementary file 1 — Additional file 1: Table 1S. Complete dataset used for the study. Adenomas evidenced in red were excluded from the statistical analysis since they did not match the selection criteria. Abbreviations : M = male, F = female, CNTRL = control, TB = tubular, TBV = tubulovillous, V = villous, mm= millimeter, SX = left, DX = right. [file 13148_2021_1135_MOESM1_ESM.pdf]

PATIENT ID	ADENOMA ID	SEX	AGE	GROUP	YEAR ADENOMA	HYSTOLOGY	DYSPLASIA GRADE	DIAMETER (mm)	N° POLYPS	LOCALIZATION	MUTATIONAL STATUS	YEAR CRC	MONTHS FREE FROM CRC	L1 Methylation %	L1-MET Methylation %
S1	A1	M	62	CNTRL	2001	TBV	HIGH	18	2	sx	KRAS p.G12D c.35G>A		216	66,53	65,13
S2	A2	F	66	CNTRL	2000	TB	LOW	6	3	sx	WT		228	61,38	65,60
S3	A3	F	66	CNTRL	2000	TBV	LOW	18	1	sx	WT		228	59,48	62,37
S4	A4	F	77	CNTRL	2001	TBV	HIGH	18	1	sx	WT		216	60,88	63,03
S5	A5	F	54	CNTRL	2001	TBV	HIGH	7	2	sx	WT		216	63,03	66,40
S6	A6	M	82	CNTRL	2001	TBV	LOW	18	1	sx	WT		216	62,03	65,67
S7	A7	F	75	CNTRL	2000	TBV	LOW	5	1	sx	WT		228	62,03	66,03
S8	A8	M	72	CNTRL	2000	TBV	LOW	10	3	sx	KRAS p.G12V c.35G>T		228	59,05	66,37
S9	A9	M	50	CNTRL	2003	TBV	LOW	5	2	dx	KRAS p.G12D c.35G>A		192	60,55	63,43
S10	A10	M	67	CNTRL	2015	TB	LOW	5	2	dx	WT		48	62,40	61,20
S11	A11	M	68	CNTRL	2015	TB	LOW	5	4	dx	WT		48	60,05	59,00
S12	A12	F	84	CNTRL	2015	TB	LOW	5	2	dx	WT		48	63,13	62,90
S13	A13	M	59	CNTRL	2015	TB	LOW	5	3	dx	WT		48	60,38	62,23
	A14				2015	TB	LOW	5	3	dx	WT			60,73	62,56
	A15				2015	TB	LOW	NA	3	sx	WT			61,06	62,06
S14	A16	M	57	CNTRL	2015	TB	LOW	5	4	sx	WT		48	60,07	60,36
	A17				2015	TB	LOW	5	4	sx	WT			62,75	60,08
S15	A18	F	61	CNTRL	2015	TB	LOW	5	2	dx	WT		48	67,30	68,60
S16	A19	F	61	CNTRL	2015	TB	LOW	5	6	dx	KRAS p.G12A c.35G>C		48	62,90	67,00
S17	A20	M	73	CNTRL	2015	TB	LOW	5	2	NA	WT		48	70,00	65,03
S18	A21	F	74	CNTRL	2003	TB	LOW	5	2	sx	WT		192	69,23	65,07
S19	A22	M	68	CNTRL	2005	TBV	LOW	9	1	sx	KRAS p.G12E c.35_36delGTinsAA		168	60,88	58,40
S20	A23	F	64	CNTRL	2005	TBV	LOW	12	1	sx	WT		168	59,43	56,93
S21	A24	F	57	CNTRL	2005	TBV	LOW	5	1	sx	WT		168	65,43	60,47
S22	A25	F	52	CNTRL	2006	TBV	LOW	12	1	sx	WT		156	58,10	57,57
S23	A26	M	64	CNTRL	2006	TBV	LOW	6	1	dx	WT		156	59,60	58,87
S24	A27	M	73	CNTRL	2004	TBV	LOW	15	2	sx	WT		180	62,45	60,37
	A28				2004	TBV	LOW	10	2	sx	WT			59,13	61,97
S25	A29	F	61	CNTRL	2006	TBV	LOW	10	1	sx	KRAS p.G12S c.34G>A		156	62,08	58,57
S26	A30	M	38	CNTRL	2006	TBV	HIGH	10	1	dx	KRAS p.G13D c.38G>A		156	56,60	56,27
S27	A31	M	44	CNTRL	2012	TB	HIGH	16	1	dx	KRAS p.G13D c.38G>A		84	53,80	55,77
S28	A32	M	50	CNTRL	2006	TBV	LOW	6	1	dx	WT		156	60,33	57,47
S29	A33	M	69	CNTRL	2004	TBV	LOW	20	1	sx	WT		180	58,33	57,40
S30	A34	M	64	CNTRL	2008	TBV	LOW	22	2	dx	KRAS p.G12D c.35G>A		132	57,25	54,50
	A35				2008	TBV	LOW	22	2	dx	KRAS p.G12D c.35G>A			58,20	55,90
S31	A36	F	49	CNTRL	2008	TBV	LOW	5	1	sx	WT		132	60,40	58,30
S32	A37	M	63	CNTRL	2008	TBV	LOW	5	1	dx	WT		132	59,30	58,00
S33	A38	F	75	CNTRL	2008	TBV	LOW	20	2	sx	KRAS p.G13D c.38G>A		132	58,63	55,90
	A39				2008	TBV	LOW	20	2	sx	KRAS p.G13D c.38G>A			60,10	56,50
S34	A40	F	59	CNTRL	2008	TB	LOW	5	1	sx	WT		132	61,75	57,40
S35	A41	F	74	CNTRL	2010	TBV	LOW	5	2	dx	NRAS p.G12D c. 35G>A		108	53,38	50,10
	A42				2010	TB	LOW	5	2	dx	WT			67,60	53,40
S36	A43	F	66	CNTRL	2010	TB	LOW	5	2	sx	WT		108	58,10	58,30
S37	A44	F	76	CNTRL	2010	TBV	HIGH	30	1	sx	KRAS p.G12D c.35G>A		108	54,00	47,20
	A45				2010	TBV	HIGH	30	1	sx	KRAS p.G12D c.35G>A			56,70	51,20
	A46				2010	TBV	HIGH	30	1	sx	KRAS p.G12D c.35G>A			55,80	48,80
	A47				2010	TBV	HIGH	30	1	sx	KRAS p.G12D c.35G>A			54,00	47,20
S38	A48	F	70	CNTRL	2010	TB	LOW	10	1	dx	WT		108	59,10	57,10
S39	A49	M	55	CNTRL	2009	TBV	HIGH	15	2	sx	WT		120	57,90	57,50
S40	A50	M	63	CNTRL	2007	TBV	LOW	8	1	sx	WT		144	57,60	57,70
S41	A51	M	59	CNTRL	2007	TBV	LOW	11	1	sx	WT		144	60,00	59,10
S42	A52	M	64	CNTRL	2007	TB	LOW	13	1	sx	KRAS p.A146T c.436G>A		144	56,60	56,10
S43	A53	M	62	CNTRL	2007	TBV	HIGH	15	1	sx	KRAS p.G12V c.35G>T		144	77,00	59,70
S44	A54	F	65	CASE	2006	TBV	LOW	15	1	sx	WT	2012	72	58,20	57,93
S45	A55	M	60	CASE	2003	TBV	HIGH	30	3	sx	KRAS p.G13D c.38G>A	2012	108	55,18	54,23
S46	A56	M	72	CASE	2005	TBV	HIGH	18	3	dx	KRAS p.G12A c.35G>C	2016	132	61,28	59,83
	A57				2005	TBV	HIGH	30	3	dx	KRAS p.G12A c.35G>C			62,70	60,87
S47	A58	M	58	CASE	2006	TBV	HIGH	13	1	dx	WT	2014	96	59,23	60,83
S48	A59	F	76	CASE	2007	TBV	LOW	13	3	sx	WT	2015	96	67,90	58,87
	A60				2007	TB	LOW	20	3	dx	WT			57,50	59,43
S49	A61	M	78	CASE	2011	TBV	LOW	20	2	dx	KRAS p.G12D c.35G>A	2015	48	63,80	59,27
	A62				2011	TB	LOW	20	2	dx	KRAS p.G12D c.35G>A			57,10	61,93
S50	A63	M	67	CASE	2011	TB	LOW	20	1	sx	WT	2015	48	65,78	59,47

S51	A64	F	82	CASE	2012	TB	LOW	15	1	sx	KRAS p.G12D c.35G>A	2015	36	69,25	62,97
S52	A65	F	69	CASE	2013	TB	LOW	NA	1	sx	WT	2014	12	65,68	60,30
S53	A66	F	68	CASE	2006	TBV	LOW	23	1	dx	KRAS p.G13D c.38G>A	2014	96	49,75	52,43
S54	A67	F	82	CASE	2008	TB	LOW	20	3	dx	WT	2014	72	55,55	59,03
S55	A68	M	66	CASE	2010	TB	LOW	20	2	dx	WT	2016	72	51,30	56,00
S56	A69	M	61	CASE	2011	TB	LOW	20	2	sx	WT	2014	36	59,70	57,23
S57	A70	M	74	CASE	2013	TB	LOW	6	1	sx	WT	2015	24	61,20	59,20
S58	A71	M	69	CASE	2013	TBV	LOW	20	4	dx	KRAS p.G12A c.35G>C	2016	36	58,20	56,80
S59	A72	M	63	CASE	2014	TB	LOW	20	1	sx	WT	2015	12	67,00	62,20
S60	A73	M	72	CASE	2015	TB	LOW	20	1	sx	WT	2016	12	63,50	57,70
S61	A74	M	73	CASE	2015	TBV	HIGH	55	2	sx	KRAS p.G12V c.35G>T	2016	12	49,70	56,80
	A75				2015	V	HIGH	15	2	sx	KRAS p.G12V c.35G>T		12	52,63	58,07
S62	A76	F	58	CASE	2000	TBV	LOW	NA	1	sx	KRAS p.G13D c.38G>A	2014	168	55,40	57,47
S63	A77	M	60	CASE	2009	TB	LOW	20	3	sx	BRAF p.V600E c.1799T>A	2011	24	63,20	59,80
S64	A78	F	68	CASE	2004	TBV	HIGH	11	1	sx	KRAS p.G13D c.38G>A	2011	84	59,10	59,80
S65	A79	M	79	CASE	2003	TB	LOW	7	1	sx	KRAS p.G13C c.37G>T	2011	96	60,30	59,20
	A80				2006	TB	LOW	20	2	dx	WT		60	50,90	ND
S66	A81	F	62	CASE	2010	TBV	LOW	10	1	sx	WT	2011	12	59,70	59,80
S67	A82	F	79	CASE	2006	TB	LOW	20	2	dx	WT	2011	60	50,90	ND
S68	A83	F	79	CASE	2006	TB	LOW	21	2	sx	WT	2011	60	54,20	ND
S69	A84	M	81	CASE	2005	TB	LOW	10	1	sx	WT	2011	72	56,80	ND
S70	A85	F	82	CASE	2005	TBV	HIGH	20	1	sx	KRAS p.G12C c.34G>T	2011	72	50,20	ND
S71	A86	M	71	CASE	1998	TBV	LOW	27	3	sx	KRAS p.G12C c.34G>T	2012	168	53,90	55,50
	A87				1998	TBV	LOW	NA	3	sx	WT			53,00	55,50
	A88				1998	TBV	LOW	NA	3	sx	WT			52,30	54,60
S72	A89	M	74	CASE	2008	TB	LOW	20	1	dx	WT	2012	48	56,70	60,00
S73	A90	F	61	CASE	2009	TB	LOW	10	1	sx	WT	2012	36	56,60	58,10
S74	A91	M	69	CASE	2007	TB	LOW	20	3	dx	WT	2010	36	57,60	ND
	A92				2007	TB	LOW	20	3	sx	WT			60,40	ND
S75	A93	M	70	CASE	2007	TB	LOW	20	1	dx	WT	2012	60	52,50	ND
S76	A94	M	81	CASE	2008	TB	LOW	22	2	dx	NA	2012	48	59,10	ND
S77	A95	M	54	CASE	2008	TB	LOW	21	1	dx	WT	2012	48	53,60	ND
S78	A96	M	71	CASE	2007	TBV	LOW	5	3	sx	BRAF p.V600E c.1799T>A	2015	96	61,95	ND
S79	A97	M	66	CASE	2004	TB	LOW	10	2	sx	PIK3CA p.E545K c.1633G>A	2017	156	65,13	60,50
	A98				2004	TB	LOW	10	2	sx	PIK3CA p.E545K c.1633G>A			65,13	60,50
S80	A99	M	64	CASE	2000	TB	HIGH	10	1	sx	WT	2003	36	66,23	59,40
S81	A100	F	67	CASE	2006	TBV	LOW	5	1	dx	WT	2015	108	63,88	ND
S82	A101	F	67	CASE	2012	TB	LOW	5	3	dx	WT	2016	48	61,65	ND
S83	A102	M	75	CASE	2012	TBV	LOW	14	1	sx	KRAS p.G12A c.35G>C	2017	60	56,05	54,60

In red adenomas excluded from the statistical analysis, as you can see in Methods (Selection Criteria and clinical-pathological analysis of adenomas)
