# Supplementary figures and images for: Evaluation of global and intragenic hypomethylation in colorectal adenomas improves patient stratification and colorectal cancer risk prediction
Source: Clin Epigenetics. 2021 Aug 9;13:154. doi: 10.1186/s13148-021-01135-0 (PMC8351348; doi:10.1186/s13148-021-01135-0)

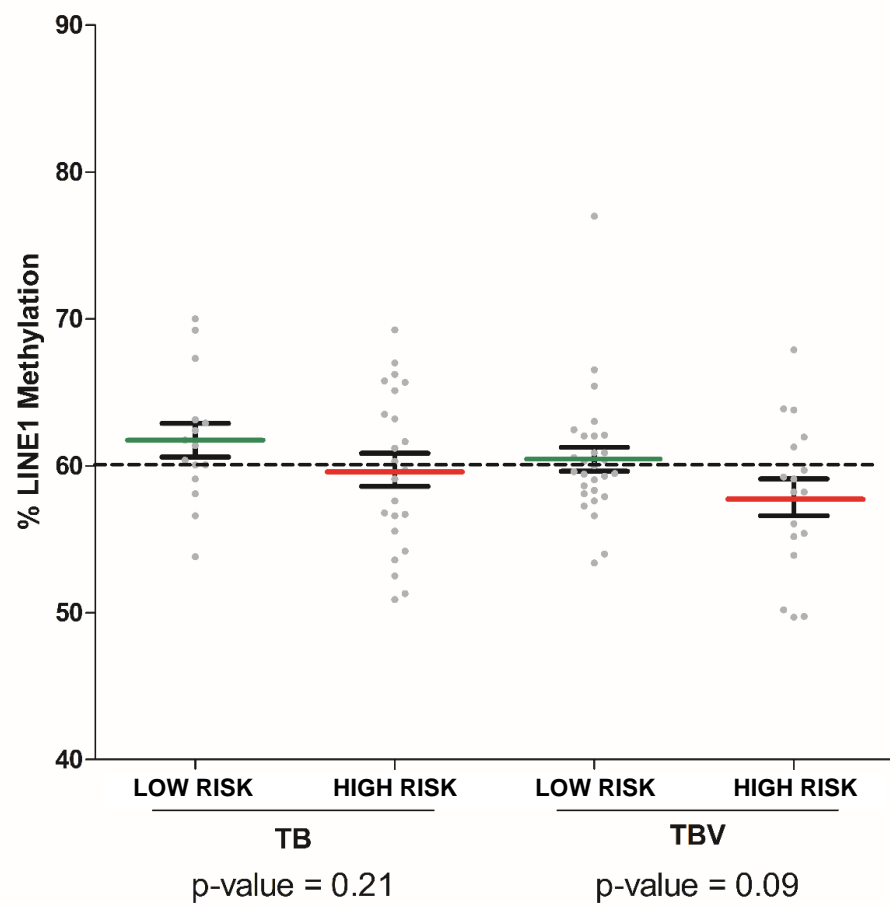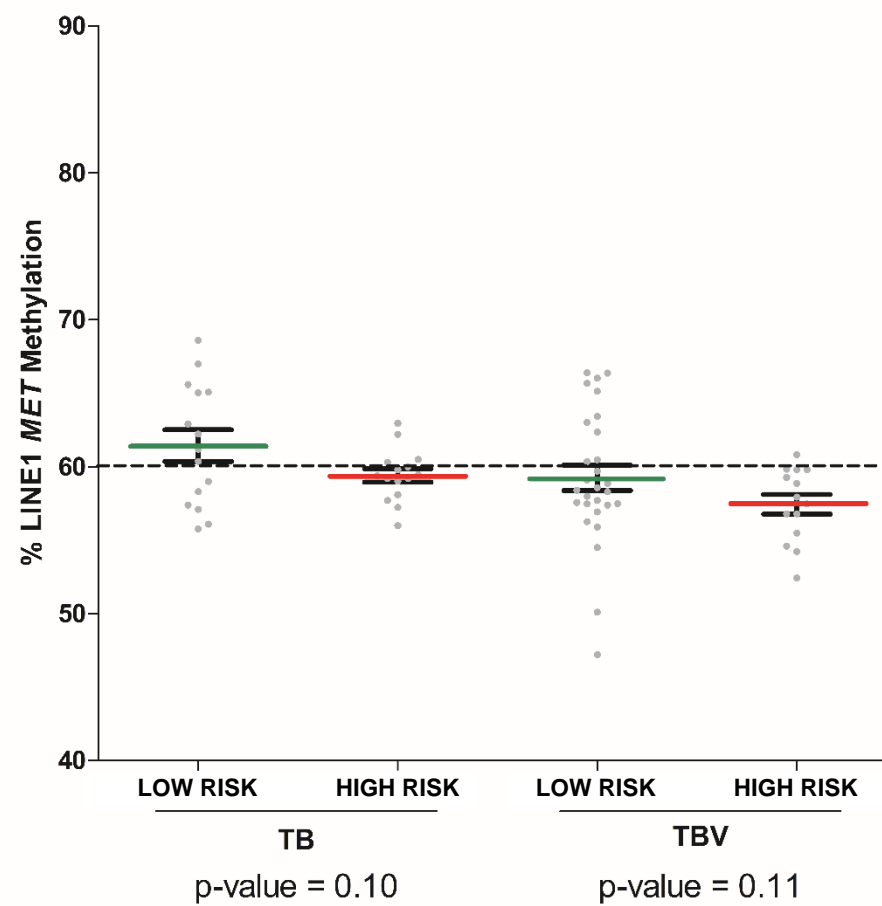

Supplement: Supplementary file 2 — Additional file 2: Fig. S1. Methylation level in relation to the adenoma histotype. A) LINE-1 methylation level in tubular and tubulovillous/villous adenomas divided in cases and controls (CNTRL). B) L1-MET methylation level in tubular and tubulovillous/villous adenomas divided in cases and controls (CNTRL). The dotted line indicates the methylation threshold (60%) below which adenomas are considered hypomethylated. The green lines indicate the average level of methylation of the low-risk group, while the red lines indicate the average level of high-risk group. [file 13148_2021_1135_MOESM2_ESM.pdf]
